# Predictive Factors of Acute Symptomatic Seizures in Patients With Ischemic Stroke Due to Large Vessel Occlusion

**DOI:** 10.3389/fneur.2022.894173

**Published:** 2022-05-27

**Authors:** Lisa Marie Tako, Adam Strzelczyk, Felix Rosenow, Waltraud Pfeilschifter, Helmuth Steinmetz, Rejane Golbach, Jan Hendrik Schäfer, Johann Philipp Zöllner, Konstantin Kohlhase

**Affiliations:** ^1^Department of Neurology, Epilepsy Center Frankfurt Rhine-Main, University Hospital Frankfurt, Goethe University Frankfurt, Frankfurt, Germany; ^2^LOEWE Center for Personalized and Translational Epilepsy Research, Goethe University Frankfurt, Frankfurt, Germany; ^3^Department of Neurology and Neurophysiology, Lüneburg Hospital, Lüneburg, Germany; ^4^Institute of Biostatistics and Mathematical Modelling, University Hospital Frankfurt, Goethe University Frankfurt, Frankfurt, Germany

**Keywords:** epilepsy, seizure, intravenous thrombolysis, mechanical recanalization, stroke unit

## Abstract

**Introduction:**

Acute symptomatic seizures (ASz) after ischemic stroke are associated with increased mortality; therefore, identifying predictors of ASz is important. The purpose of this study was to analyze predictors of ASz in a population of patients with ischemic stroke due to large arterial vessel occlusion (LVO).

**Materials and Methods:**

This retrospective study examined patients with acute ischemic stroke caused by LVO between 2016 and 2020. Identification of predictive factors was performed using univariate and subsequent multiple logistic regression analysis. In addition, subgroup analysis regarding seizure semiology and time of seizure occurrence (≤ 24 h and > 24 h after stroke) was performed.

**Results:**

The frequency of ASz among 979 patients was 3.9 % (*n* = 38). Univariate logistic regression analysis revealed an increased risk of ASz in patients with higher National Institutes of Health Stroke Scale (NIHSS) score at admission or 24 h after admission, hypernatremia at admission ≥ 145 mmol/L, and pneumonia. Further multiple logistic regression analysis revealed that NIHSS 24 h after admission was the strongest predictor of ASz, particularly relating to ASz occurring later than 24 h after stroke. Patients who experienced a seizure within the first 24 h after stroke were more likely to have a generalized tonic-clonic (GTCS) and focal motor seizure; beyond 24 h, seizures with impaired awareness and non-convulsive status epilepticus were more frequent.

**Conclusion:**

NIHSS score 24 h after admission is a strong predictive factor for the occurrence of ASz in patients with ischemic stroke caused by LVO. The semiology of ASz varied over time, with GTCS occurring more frequently in the first 24 h after stroke.

## Introduction

Cerebrovascular disease is the most common cause of epilepsy in the elderly, accounting for up to 39–49% of all newly diagnosed epilepsies in patients aged > 60 years (1, 2). Due to demographic changes, the incidence of stroke-related epilepsy is expected to rise and pose an increasing challenge for the healthcare system (3). Depending on the time course, seizures after stroke are defined according to the International League Against Epilepsy (ILAE) either as an acute symptomatic seizure (ASz) if they occur within 7 days, or as an unprovoked late seizure if they occur later than 7 days (4). Acute symptomatic seizures are thought to result from local cellular biochemical dysfunction of electrically excitable tissues, whereas late seizures are caused by post-ischemic remodeling of the damaged brain tissue and neuronal network, leading to an acquired predisposition to seizures and the diagnosis of post-stroke epilepsy (5–8). A large systemic review and meta-analysis examined the frequency of seizures after ischemic stroke; the frequency of ASz was found to be 3.3% and the late post-stroke seizure frequency was 1.8% (9). Because ASz are associated with an increased risk of mortality, knowledge of predictive factors is essential (10, 11). Various risk factors with different levels of evidence are described in the literature. The severity of stroke, estimated by the National Institutes of Health Stroke Scale (NIHSS), and cortical involvement were identified as independent risk factors for the occurrence of ASz (11–16). Data are inconclusive regarding other possible risk factors, such as cardioembolic infarct etiology, anterior circulation cerebral infarction, hemorrhagic transformation, previous transient ischemic attack (TIA), acute non-neurological infection, and history of diabetes mellitus (10, 11, 13, 17). Based on these results, different prediction models have been developed to assess the individual risk for post-stroke seizures (18, 19). Furthermore, therapy with statins in the acute phase of stroke was reported to reduce the rate of seizures (20). Systemic thrombolysis and mechanical thrombectomy as established reperfusion procedures have also been the subject of research, with recent studies showing no association with ASz frequency (21, 22).

The variability among identified predictive factors may be explained by the heterogeneous designs of the available studies, with varying levels of evidence (registry studies, retrospective and prospective designs, mono- or multi-centric studies, systematic reviews), inclusion criteria (hemorrhagic and ischemic stroke), and definitions of ASz occurring later than 7 days (17, 23, 24). Furthermore, the studies were conducted over an extended period, including several studies in which neurological treatment in stroke units differed and new therapeutic milestones, such as mechanical recanalization, had not yet been established.

The purpose of this study is to analyze predictive factors for ASz in a well-defined patient population who experienced an ischemic stroke due to large vessel occlusion (LVO) and who were treated after mechanical recanalization had become the standard therapy for LVO in 2016.

## Materials and Methods

This study analyzed data from patients with acute ischemic stroke who were treated at the University Hospital Frankfurt between 2016 and 2020. The Local Ethics Committee of Goethe University Frankfurt approved the study (IRB: 19-285). Due to the retrospective design of this study, the requirement for written informed consent of the patients was waived. Strengthening the Reporting of Observational Studies in Epidemiology (STROBE) guidelines were closely followed (25).

We included patients with a final International Statistical Classification of Diseases and Related Health Problems, 10th revision (IDC-10) diagnosis of “acute ischemic stroke caused by an occlusion of a large cerebral vessel.” The occlusion of a large cerebral vessel was confirmed either by imaging with CT or MR angiography, or by infarct demarcation that could only be explained by a proximal vessel occlusion. LVO was defined according to the literature as an occlusion of the internal carotid artery, middle cerebral artery (including M1 and M2 segments), anterior cerebral artery (including A1 and A2 segments), posterior cerebral artery (including P1 and P2 segments), vertebral artery, or basilar artery. LVOs were further divided into either anterior (internal carotid artery, middle cerebral artery, and anterior cerebral artery) or posterior (posterior cerebral artery, vertebral artery, and basilar artery) circulation.

The collected data contained the following variables: age; sex; NIHSS score at admission, 24 h after admission, and at discharge; modified Rankin Scale (mRS) score prior to stroke and at discharge; type of LVO; stroke etiology according to the Trial of ORG 10172 in Acute Stroke Treatment (TOAST) criteria (26); history of arterial hypertension, diabetes mellitus, atrial fibrillation, chronic alcohol consumption, previous stroke, and brain tumor; known neurodegenerative disease; previous treatment with oral anticoagulants, antiplatelet agents, or statins; serum level of sodium (mmol/L), glucose (mg/dl), and total cholesterol (mg/dl) at admission; and hemorrhagic transformation. In addition, preexisting treatment with anti-seizure medication (ATC code N03A irrespective of indication, e.g., mood stabilizer or management of neuropathic pain) was ascertained. Furthermore, treatment with mechanical thrombectomy or systemic thrombolysis was assessed. In patients who underwent mechanical thrombectomy, the outcome of mechanical recanalization was reported according to the TICI (thrombolysis in cerebral infarction) grading system, with a score of at least 2b considered successful reperfusion (27). Mechanical thrombectomy is routinely performed under general anesthesia with subsequent weaning on the intensive care unit. The occurrence of pneumonia during hospital stay was assessed, which was defined by radiological evidence of pneumonia-compatible findings (e.g., infiltrates, consolidation) with associated clinical (e.g., purulent sputum, hypoxia, dyspnea, tachypnea, pathologic auscultation of the lungs, fever > 38.3°C) and laboratory signs (e.g., leukopenia < 4,000 leukocytes/mm^3^, leukocytosis > 12,000/mm^3^).

An ASz was defined as either clinically observed ictal stigmas or subclinical seizure patterns or non-convulsive status epilepticus (NCSE) recorded on electroencephalography (EEG) within the first 7 days after stroke (seizure latency) (4). EEG was performed according to the indication of the attending physician; standard continuous EEG diagnostics was not performed. There was no prophylactic antiseizure medication administered after ischemic stroke. Further subgroup analysis was performed for seizures occurring ≤ 24 h and later than 24 h until 7 days of stroke onset. Based on the reported semiology and EEG patterns, seizures were subdivided into: (I) seizures with impaired awareness or NCSE, (II) focal motor seizures, and (III) generalized tonic-clonic seizures (GTCS) (28).

For the regression analysis, we further divided the numerical and ordinally scaled variables into categories. NIHSS score was divided into three groups: 0-5 points, corresponding to none or mild neurological deficit; 6-15 points, corresponding to moderate neurological deficit; and more than 15 points, corresponding to severe neurological deficit. We also divided the mRS score into two groups: 0-2 points for patients with no significant or only slight disability in their daily lives, and 3-5 points for patients with moderate-to-severe disability. Furthermore, sodium level at admission was classified as hyponatremia (≤ 135 mmol/L), normal (136–144 mmol/L), or hypernatremia (≥ 145 mmol/L). The glucose level was classified as hypo- (≤ 50 mg/dl) or hyperglycemia (≥ 200 mg/dl).

Statistical analyses were performed using SPSS (version 27.0.1.0, IBM Corp., Armonk, NY, USA). For statistical evaluation, univariate binary logistic regression analysis was initially performed for independent variables, with ASz as the dependent variable. Based on this analysis, significant (*p*-value < 0.1) independent variables were selected for further multiple binary logistic regression analysis. Due to a total of 38 ASz, the number of covariates to be included was limited to 4 variates (the number of positive cases in the dependent variable divided by 10). Intergroup differences in seizure semiology regarding time of occurrence were calculated using the chi-squared test. *P*-values were corrected for multiple testing by using the Benjamini–Hochberg false discovery rate method. For multiple logistic regression and the chi-squared test, a *p*-value < 0.05 was determined to be significant.

## Results

A total of 979 patients were included in this study, with an ASz frequency of 3.9 % (*n* = 38). For an overview of patient characteristics and the results of the univariate binary logistic regression analysis, (see Table 1). Among the evaluated independent variables, univariate binary logistic regression analysis revealed an increased risk of ASz in patients with a NIHSS score > 15 points at admission (47.4 vs. 33.7%, *p* = 0.09), 24 h after admission (47.8 vs. 23.5%, *p* = 0.025) and at discharge (33.3 vs. 9.5%, *p* = 0.021), which was also found for the numeric NIHSS score at admission, after 24 h and at discharge. Furthermore, patients with ASz were more likely than patients without ASz to have hypernatremia at admission (18.4 vs. 6.5%, *p* = 0.048) or pneumonia (47.4 vs. 29.4%, *p* = 0.07). A mRS score at discharge of 3-5 points was significantly more common in patients who suffered an ASz than in those who did not (87.0 vs. 49.3%, *p* = 0.017), which corresponds to greater disability in these patients after stroke.

**Table 1:** Univariate logistic regression analysis of clinical parameters and their association with acute symptomatic seizures in patients after ischemic stroke.

**Predictor**		**Number of available data; %**	**Acute symptomatic seizure, *n* = 38**	**No acute symptomatic seizure, *n* = 941**	**Corrected *p*-value**
Age	Mean ± SD	979; 100%	72.3 ± 11.6	71.0 ± 14.0	0.87
Female gender		979; 100%	17; 44.7%	453; 48.1%	0.89
NIHSS at admission	≤ 5*	978; 99.9%	4; 10.5%	261; 27.8%	0.18
	6–15		16; 42.1%	362; 38.5%	0.17
	>15		18; 47.4%	317; 33.7%	**0.09**
	Median (Q1–Q3)		15 (9–21)	12 (5–17)	**0.029**
NIHSS after 24 h	≤ 5*	754; 77.0%	4; 17.5%	340; 46.5%	**0.09**
	6–15		8; 34.8%	219; 30.0%	0.19
	>15		11; 47.8%	172; 23.5%	**0.025**
	Median (Q1–Q3)		15 (6–24)	6 (2–15)	**0.043**
NIHSS at discharge	≤ 5*	607; 62.0%	5; 27.8%	399; 67.7%	**0.02**
	6–15		7; 38.9%	134; 22.8%	**0.08**
	>15		6; 33.3%	56; 9.5%	**0.021**
	Median (Q1–Q3)		11.5 (3.75–20.25)	2 (0–8)	**0.014**
mRS at admission	0-2*	944; 96.4%	30; 81.1%	764; 84.2%	0.87
	3-5		7; 18.9%	143; 15.8%	
mRS at discharge	0-2*	629; 64.2%	3; 13.0%	307; 50.7%	**0.017**
	3-5		20; 87.0%	299; 49.3%	
Vascular territory	Anterior circulation*	979; 100%	34; 89.5%	770; 81.8%	0.32
	Posterior circulation	979; 100%	3; 7.9%	158; 16.8%	0.17
	Both circulations	979; 100%	1; 2.6%	13; 1.4%	0.6
Etiology	Unknown or ESUS*	978; 99.9%	12; 31.6%	206; 21.9%	0.91
	Atherothrombotic		10; 26.3%	323; 34.4%	0.3
	Cardioembolic		16; 42.1%	367; 39.0%	0.9
	Other		0; 0.0%	44; 4.7%	1.0
Acute stroke therapy	ST	979; 100%	17; 44.7%	413; 43.9%	1.0
	MT	979; 100%	19; 50.0%	453; 48.1%	0.93
	ST and MT	979; 100%	12; 31.6%	252; 26.8%	0.88
	MT with TICI ≥ 2b	472; 100%	17; 89.5%	386; 85.2%	0.85
Preexisting therapies	Oral anticoagulant	979; 100%	5; 13.2%	144; 15.3%	0.88
	Antiplatelet	979; 100%	11; 28.9%	302; 32.1%	0.86
	Statin	979; 100%	14; 36.8%	250; 26.6%	0.34
	Anti-seizure medication	979; 100%	1; 2.6%	61; 6.5%	0.7
Blood glucose level at admission	≤ 50 mg/dl	954; 97.4%	1; 2.6%	2; 0.2%	0.15
	≥200 mg/dl		2; 5.3%	82; 9.0%	0.82
Sodium level at admission	≤ 135 mmol/l	966; 98.7%	2; 5.3%	80; 8.6%	0.89
	≥145 mmol/L		7; 18.4%	60; 6.5%	**0.048**
Total cholesterol level > 200 mg/dl		763; 78%	4; 18.2%	152; 20.5%	0.93
Arterial hypertension		979; 100%	28; 73.7%	695; 73.9%	1.0
Atrial fibrillation		979; 100%	16; 42.1%	359; 38.2%	0.85
Chronic alcohol consumption		979; 100%	2; 5.3%	51; 5.4%	1.0
Neurodegenerative disease		979; 100%	3; 7.9%	53; 5.6%	0.89
Previous brain tumor		979; 100%	0; 0.0%	7; 0.7%	1.0
Previous ischemic stroke		979; 100%	11; 28.9%	177; 18.8%	0.29
Hemorrhagic transformation		979; 100%	2; 5.3%	27; 2.0%	0.75
Pneumonia		979; 100%	18; 47.4%	277; 29.4%	**0.07**

* *Indicates the reference category in regression analysis. P-values were corrected using the Benjamini–Hochberg false discovery rate method with a p-value < 0.1 considered statistically significant; significant values are marked bold. NIHSS, National Institutes of Health Stroke Scale; mRS, modified Rankin Scale; ESUS, embolic stroke of undetermined source; ST, systemic thrombolysis; MT, mechanical thrombectomy; SD, standard deviation; Q1, first quartile; Q3, third quartile*.

**Figure 1:**
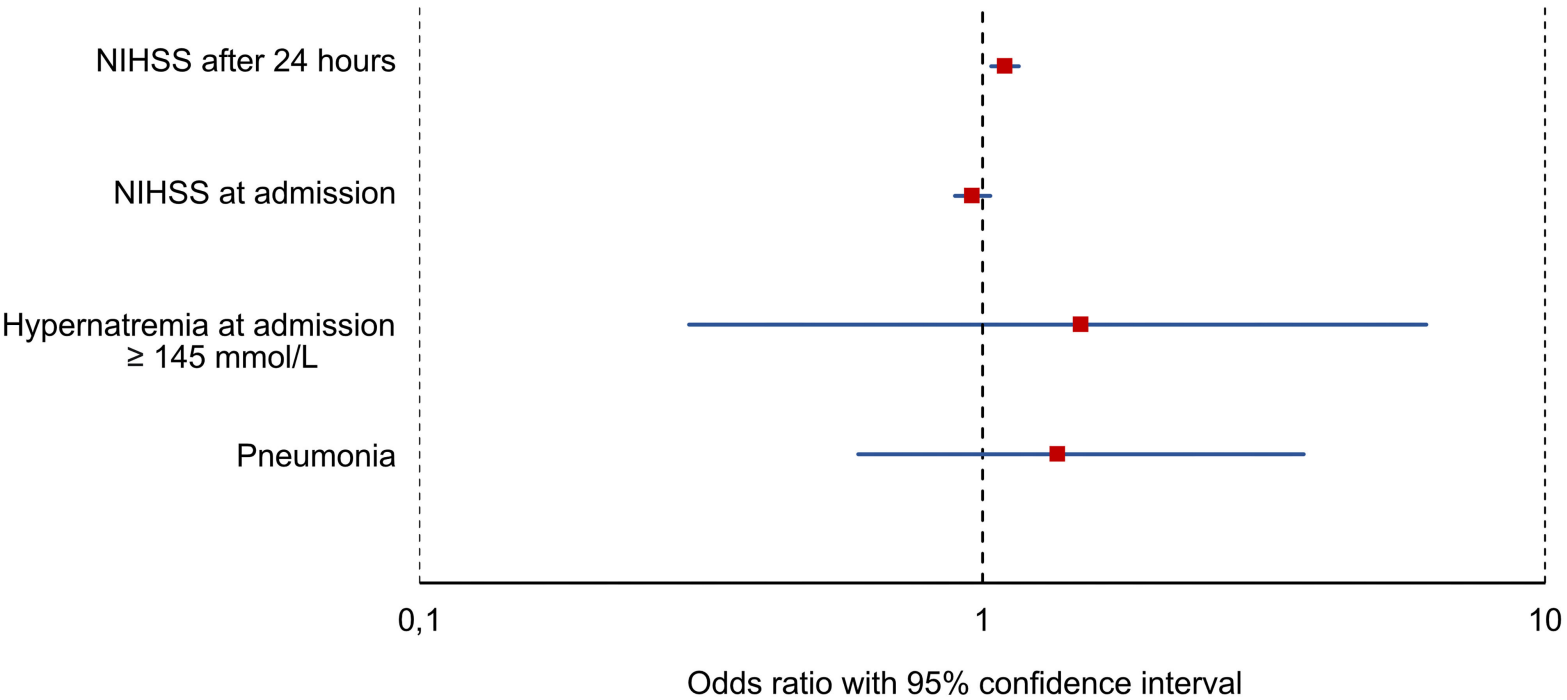
Results of the multiple regression analysis. Red squares represent the odds ratio and blue horizontal bars represent the 95% confidence interval. NIHSS, National Institutes of Health Stroke Scale.

Based on the univariate logistic regression, NIHSS score at admission (numeric), NIHSS after 24 h (numeric), hypernatremia at admission, and pneumonia were included in the final multiple logistic regression analysis. Because mRS at discharge and NIHSS score at discharge are typically ascertained beyond 7 days after stroke, they were not suitable for the prediction of ASz and therefore were not included. Following multiple logistic regression analysis, NIHSS score after 24 h was the strongest predictor for the occurrence of ASz (OR: 1.096, 95% CI: 1.036–1.159, *p* < 0.001), whereas NIHSS score at admission, pneumonia, and hypernatremia at admission did not reach the level of significance (Table 2 and Figure 1).

**Table 2:** Multiple logistic regression analysis showing the influence of different variables on seizure risk.

**Predictor**	***P*** **-value**	**Exp(B)**	**95% CI lower bound**	**95% CI upper bound**
NIHSS after 24 h	**<0.001**	1.096	1.036	1.159
NIHSS at admission	0.28	0.96	0.893	1.033
Hypernatremia ≥145 mmol/L at admission	0.69	1.360	0.301	6.154
Pneumonia	0.39	1.498	0.602	3.728

*95% CI [95% confidence interval for Exp(B)]*.

*NIHSS, National Institutes of Health Stroke Scale. A p-value of < 0.05 was determined significant; significant values are marked in bold*.

Seizures occurred at a median (minimum–maximum) of 2 (0–7) days after stroke onset. More than half (*n* = 22, 57.9 %) of the seizures occurred within the first 2 days, and over three-quarters (*n* = 29, 76.3 %) of the seizures occurred within the first 3 days. For subgroup analysis of seizures, we divided them into two groups: seizure onset within the first 24 h after stroke (*n* = 11, 28.9%), and seizure onset later than 24 h after stroke (*n* = 27, 71.1%). We performed a multiple logistic regression analysis for each subgroup and included the following variables: NIHSS score at admission (numeric), NIHSS score after 24 h (numeric), hypernatremia at admission, and pneumonia. A high NIHSS score 24 h after admission was a risk factor only for the occurrence of seizures with onset later than 24 h after stroke (OR: 1.103, 95 % CI: 1.035–1.174, *p* = 0.002). The other variables had no significant influence on stroke occurrence in the subgroup analysis (Table 3). Regarding seizure semiology, significantly more GTCSs (54.5 vs. 14.8%, *p* = 0.028) and more focal motor seizures (36.4 vs. 7.4%, *p* = 0.047) occurred in the subgroup of patients who had their seizure within the first 24 h after stroke. In contrast, there were significantly more seizures with impaired awareness in the subgroup of patients who suffered from ASz later than 24 h after stroke (77.8 vs. 9.1%, *p* = 0.003). For details, refer to Table 4.

**Table 3:** Multiple logistic regression analysis with subgroups of seizure patients.

**Predictor**	**Acute symptomatic seizure with stroke** **latency ≤24 h**				**Acute symptomatic seizure with** **stroke latency > 24 h**			
	***P*** **-value**	**Exp(B)**	**95% CI lower bound**	**95% CI upper bound**	***P*** **-value**	**Exp(B)**	**95% CI lower bound**	**95% CI upper bound**
NIHSS after 24 h	0.224	1.066	0.962	1.182	**0.002**	1.103	1.035	1.174
NIHSS at admission	0.571	1.040	0.909	1.189	0.150	0.940	0.865	1.022
Hypernatremia ≥ 145 mmol/L at admission	0.366	2.755	0.306	24.820	0.952	0.939	0.119	7.402
Pneumonia	0.309	0.319	0.035	2.884	0.157	2.101	0.752	5.870

*95% CI [95% confidence interval for Exp(B)]*.

*NIHSS, National Institutes of Health Stroke Scale. A p-value of < 0.05 was determined significant; significant values are marked in bold*.

**Table 4:** Seizure semiology in patients with acute symptomatic seizures (ASz) in relation to time of stroke.

**Seizure semiology**	**Corrected *P*-value**	**ASz with latency to stroke ≤24 h** **(*n* = 11)**	**ASz with latency to stroke > 24 h** **(*n* = 27)**
Impaired awareness (*n* = 22)	**0.003**	*n* = 1 (9.1 %)	*n* = 21 (77.8 %)
Focal motor seizure (*n* = 7)	**0.047**	*n* = 4 (36.4 %)	*n* = 2 (7.4 %)
GTCS (*n* = 10)	**0.028**	*n* = 6 (54.5 %)	*n* = 4 (14.8 %)

*Impaired awareness includes non-convulsive status epilepticus (NCSE). P-values were corrected using the Benjamini–Hochberg false discovery rate method and p < 0.05 was considered statistically significant; significant values are marked in bold. GTCS, generalized tonic-clonic seizure*.

## Discussion

The aim of this study was to investigate predictors of ASz in a well-defined study population of patients with LVO treated after the paradigm shift in stroke therapy to the widespread use and availability of mechanical thrombectomy (29). In our analysis, NIHSS score after 24 h was shown to be the strongest predictor for the occurrence of ASz in patients with ischemic stroke due to LVO. A higher NIHSS score correlates with a larger volume of damaged brain tissue and concomitant cortical involvement, which can explain the increased seizure risk (30). Our study showed a stronger predictive value of the NIHSS score after 24 h compared with the NIHSS score at admission. Furthermore, in the subgroup analysis, we demonstrated that a high NIHSS score was particularly associated with the occurrence of seizures later than 24 h after stroke.

In contrast, no significant association was found between the NIHSS score and the occurrence of seizures within the first 24 h after stroke. In the literature, it was described that most seizures occurred within the first 24 h after stroke (31). This finding could not be reproduced in our study, but there were significant differences regarding seizure semiology. Significantly more focal motor seizures or GTCSs occurred within 24 h after stroke; beyond that period, seizures with reduced consciousness or NCSEs were more common. This observation could be explained by studies that did not include EEGs in their analysis, which may have led to an underestimation of the frequency of non-motor seizures or NCSEs and a predominance of motor seizures, which were more likely to occur in the first 24 h. However, besides differences in seizure semiology, ASz in our study showed a predominance within the first 48 h, which may be explained by different pathophysiological processes of ASz. Within the first hours, acute neuronal excitability due to ischemic disturbance of cellular integrity and pericellular milieu (i.e., electrolytes and neurotransmitters) may represent the prominent factor in seizure development, whereas subsequent tissue destruction and necrotic remodeling processes with surrounding inflammation could be responsible for the development of ASz beyond the first 24 h (32). This supports the hypothesis that in ASz occurring within 24 h after vascular occlusion, the extent of infarction is less important than the acute disturbances of cerebral integrity, for which a possible predisposition of the patient might be relevant. However, an increased risk in patients with a previous stroke, chronic alcohol consumption, previous brain tumor or a neurodegenerative disease was not observed. In this context, it would be of interest to investigate to what extent the risk of post-stroke epilepsy differs regarding the time of occurrence after stroke. Hemorrhagic transformation has been reported to be associated with an increased risk of ASz due to the deposition of blood degradation products such as hemosiderin, leading to local irritation of the brain (16). While hemorrhagic transformations have been consistently reported as an independent predictor of ASz (16, 33), this was not demonstrated in our study, although there was a tendency toward higher incidences of ASz in those patients. A possible reason for the missing of statistical significance might be the overall low incidence of hemorrhagic transformations in the included cohort in comparison to other trials (34).

It has been established that electrolyte imbalances are associated with ASz, with low sodium levels being particularly significant (35). Although hyponatremia seems to be more frequently associated with ASz, our study found an increased risk of ASz in patients with hypernatremia at admission (35). In particular, hypernatremia with a rapid onset, in contrast to chronic hypernatremia, is reported to show a significantly increased risk of ASz (35). Since hypernatremia was recorded at admission, a further differentiation between acute or chronic hypernatremia was not possible. Besides initial electrolyte imbalances, critically ill patients on an intensive care unit or stroke unit bear an increased risk for the occurrence of subsequent hypernatremia (e.g., iatrogenic), which was not evaluated in our study (36). Furthermore, there was a trend toward an increased risk of seizures in patients with pneumonia in univariate regression analysis; however, this did not reach the significance level in multiple logistic regression analysis. This lack of effect might be attributed to the small sample size of those who suffered an ASz and the wide 95% confidence interval. In subgroup analysis, this tendency was particularly evident between pneumonia and the occurrence of seizures later than 24 h after stroke (OR: 2.101, 95 % CI: 0.752–5.870, *p* = 0.157). Considering the predominance of ASz within the first 48 h and the increased risk of early pneumonia in patients with LVO due to general anesthesia during mechanical thrombectomy as well as stroke-related dysphagia, all pneumonias during hospital stay without formal classification into community- or hospital-acquired (>48 h after admission) were included (37). However, a more detailed analysis of the temporal association between the occurrence of pneumonia and ASz would be of interest and might be addressed in further studies. Acute non-neurological infections have been demonstrated as a predictive factor in other studies, but these studies usually did not include exclusively pneumonia (11, 13). Apart from concomitant fever, the epileptogenic effect of infections is attributed to the fact that the resulting inflammatory response, particularly by cytokines, lowers the seizure threshold and predisposes the brain to seizures (38).

Previous studies have shown associations with pre-existing diabetes mellitus and with the presence of hyperglycemia in experimental data (13, 39). In our study, hyperglycemia was not associated with an increased risk of ASz; however, hypoglycemia ≤ 50 mg/dl showed a trend toward more ASz. Since hypoglycemia ≤ 50 mg/dl was only observed in a very small number of patients (*n* = 3), this effect needs to be evaluated in future studies. As these factors are reversible causes, knowledge of their potential risk for ASz is important. Therefore, in patients with a recent stroke after LVO, regular monitoring of blood glucose and sodium levels should be performed with the aim of achieving normoglycemia and normonatremia. In addition, pneumonia prophylaxis should be practiced in addition to early treatment initiation after pneumonia has been diagnosed.

Regarding reperfusion therapies, in addition to the established systemic thrombolysis, mechanical thrombectomy has been widely available since 2015/2016 as a therapeutic option for emergency reopening of LVO (29, 40). Among the included patients, 50% with ASz and 48.1% without ASz received mechanical thrombectomy. Initial reports suggested that these methods may lead to an increased risk of ASz due to either neurotoxicity or reperfusion damage (41, 42). However, recent studies have not confirmed this and have instead supported the protective effect of reperfusion therapies in reducing the extent of infarction (21, 22). In concordance, this study, which examined only patients with ischemic stroke due to LVO, showed no association between treatment with systemic thrombolysis or mechanical thrombectomy and the occurrence of ASz. Previous studies described an increased risk of post-stroke epilepsy in severe strokes of the anterior circulation, which was attributed to a larger tissue damage (43). In our study, vascular territory (anterior or posterior circulation) was not found to be a predictive factor of ASz, although the overall smaller number of patients with posterior circulation infarcts should be noted. Further studies with a larger number of ASz and a volumetric evaluation of infarct size and location are required to determine their influence on the occurrence of ASz.

Despite a careful evaluation of all patients, this study had several limitations. Due to the limited number of ASz cases, the possible influence of predictive factors might have been missed. This may explain why some previously described predictors did not reach statistical significance in our analysis. Nevertheless, while this study bears the risk of missing rare variables as predictors, the well-defined population of this single center study allowed us to collect data on seizure semiology and time of seizure onset; this information was often lacking in larger registry studies. The total number of ASz cases may still be underestimated without routine or continuous use of EEG, especially in intensive care units (44). Further studies with prospective designs to detect seizures by routine and continuous EEG in addition to systematic clinical examinations as well as studies with a larger sample size and long-term follow-up are necessary.

## Conclusion

A high NIHSS score 24 hours after admission is one of the most important predictive factors for the occurrence of ASz in patients following ischemic stroke, especially for ASz occurring later than 24 h after stroke. Semiology of ASz differed significantly, with focal motor seizures or GTCSs occurring more frequently within the first 24 h after stroke, whereas seizures with impaired awareness or NCSE were more frequent beyond 24 h.

## Data Availability Statement

The data analyzed in this study is subject to the following restrictions: Anonymised data will be made available after reasonable request due to german regulations on data protection.

## Ethics Statement

This retrospective study was reviewed and approved by the Ethics Committee of the Medical Faculty of the Goethe University Frankfurt. Written informed consent for was not required for this study.

## Author Contributions

LT and KK conducted the study, collected the data, and wrote the original draft of the manuscript. LT, RG, and KK carried out the statistical analysis of the data. AS, WP, and KK were responsible for the methodological conceptualization of the study. AS was responsible for the supervision of the study. HS, FR, JS, and JZ contributed to the finalization of the manuscript and the formal analysis of the data. LT, AS, FR, WP, HS, RG, JS, JZ, and KK reviewed and revised the manuscript. All authors contributed to the manuscript and accepted the final version of the manuscript.

## Conflict of Interest

AS reports personal fees and grants from Angelini Pharma, Desitin Arzneimittel, Eisai, GW Pharmaceuticals, Marinus Pharma, Precisis, UCB, UNEEG Medical, and Zogenix, outside the scope of the submitted manuscript. FR reports personal fees from Angelini Pharma/Arvelle Therapeutics, Eisai GmbH, GW Pharmaceuticals/Jazz Pharma, and UCB Pharma and grants from the Detlev-Wrobel-Fonds for Epilepsy Research, the Deutsche Forschungsgemeinschaft (DFG), the Federal Ministry of Education and Research (BMBF), the LOEWE Programme of the State of Hesse, and the European Union, outside the scope of the submitted manuscript. JZ reports speaker's honoraria from Desitin Arzneimittel, Eisai and GW Pharmaceuticals, outside the scope of the submitted manuscript. The remaining authors declare that the research was conducted in the absence of any commercial or financial relationships that could be construed as a potential conflict of interest.

## Publisher's Note

All claims expressed in this article are solely those of the authors and do not necessarily represent those of their affiliated organizations, or those of the publisher, the editors and the reviewers. Any product that may be evaluated in this article, or claim that may be made by its manufacturer, is not guaranteed or endorsed by the publisher.
